# Exploring Computational Thinking Skills Training Through Augmented Reality and AIoT Learning

**DOI:** 10.3389/fpsyg.2021.640115

**Published:** 2021-02-23

**Authors:** Yu-Shan Lin, Shih-Yeh Chen, Chia-Wei Tsai, Ying-Hsun Lai

**Affiliations:** ^1^Department of Information Science and Management Systems, National Taitung University, Taitung, Taiwan; ^2^Department of Computer Science and Information Engineering, National Taitung University, Taitung, Taiwan

**Keywords:** computational thinking, augmented reality, artificial intelligent of thing, problem solving skills, problem reasoning

## Abstract

Given the widespread acceptance of computational thinking (CT) in educational systems around the world, primary and higher education has begun thinking about how to cultivate students' CT competences. The artificial intelligence of things (AIoT) combines artificial intelligence (AI) and the Internet of things (IoT) and involves integrating sensing technologies at the lowest level with relevant algorithms in order to solve real-world problems. Thus, it has now become a popular technological application for CT training. In this study, a novel AIoT learning with Augmented Reality (AR) technology was proposed and explored the effect of CT skills. The students used AR applications to understand AIoT applications in practice, attempted the placement of different AR sensors in actual scenarios, and further generalized and designed algorithms. Based on the results of the experimental course, we explored the influence of prior knowledge and usage intention on students' CT competence training. The results show that proposed AIoT learning can increase students' learning intention and that they had a positive impact on problem solving and comprehension with AR technology, as well as application planning and design.

## Introduction

As science and technology continue to advance, their overall impact on everyday life is no longer limited to basic necessities like food, clothing, housing, and transportation; science and technology are also related to the scope of national education. With Alpha Go defeating humans in chess, artificial intelligence (AI) has quickly attracted global attention. Not only have many studies begun using AI to attempt to solve previously complex and difficult problems, AI education and programming skills have also gradually transitioned from information expertise in universities to an emerging general knowledge requirement for all citizens. For instance, former US President Barack Obama promulgated the Every Student Succeeds Act, which considers computer science to be a key academic field and general ability and encourages schools to incorporate data science into the basic curriculum. Programming education is no longer relegated to information science professionals; it is now a necessary basic skill that all citizens should possess in preparation for the near future. In addition to programming skills training, computational thinking (CT) involves the effective analysis and deconstruction of complex issues and their translation into computer programming languages, so that people can understand human problems from a computer programming perspective and implement solutions using computers (Wing, 2006, 2011; Grover and Pea, 2013). Integrating CT and programming training immerses students in science education from an early age, which will, to a certain extent, strengthen their ability to apply information science. Furthermore, it shifts the focus of traditional learning from “reading, writing, and arithmetic” skills to the cultivation of academic literacy through “doing, using, and thinking” (Yadav et al., 2017). The International Society for Technology in Education (ISTE) has also defined the standards for CT competences based on students' information age development needs—algorithmic thinking, creativity, logical thinking, and problem-solving skills—demonstrating that CT focuses not only on basic programming skills training but also on fostering students' competences in problem comprehension and solving, and system design. Therefore, CT is suitable for basic literacy and modes of thinking at different stages and in different fields, the integration of interdisciplinary applications, and various fields' teaching curricula (Qualls and Sherrell, 2010; Barr and Stephenson, 2011).

The popular AIoT technology combines the IoT with AI technology to create numerous smart applications, such as smart homes, smart enterprises, and even smart cities (Gubbi et al., 2013; Lee and Lee, 2015; Lai et al., 2021). The diverse sensing technologies of AIoT and AI programming learning are also compatible with various educational strategies in engineering education, maker learning, project-based learning, and problem-oriented learning (Navghane et al., 2016; Lensing and Friedhoff, 2018), thus enabling students to integrate sensor applications with AI algorithms in order to create different smart applications and solve practical problems. Through the learning process, students can not only practice using sensing technology and AI algorithms but also cultivate their competences in thinking about problems and teamwork (Hundhausen et al., 2013). The interdisciplinary integration of sensing technology and problem-solving goals also makes AIoT courses an educational environment capable of cultivating and improving students' CT competences. However, due to its diverse applications and complex environments, AIoT often results in students' lack of familiarity with the application fields, or it involves a flat design, which leads students to explore problems at insufficient depths and/or choose to ignore certain parts of the problem. For example, in smart agriculture, different environments and crops should be taken into account when considering deployment and the choice of sensors; failure to do so can lead to poor overall learning outcomes (Lai et al., 2019; Chen et al., 2020).

In view of the above, this study mainly discusses the impact of this kind of AR AIoT learning on CT skills training and introduces AIoT teaching methods with AR technology in order to explore their effects on the learning outcomes of AIoT courses and CT competency performance. Through the concept of AR space design, we aim to cultivate an understanding of programming structure and expand scientific education field data, thereby laying the foundation for students' basic science knowledge and understanding of the structure of different programming components. Students used the AR application in different fields to place the IoT sensing modules within the actual application field, which enabled them to think about and plan a suitable project design for the specific fields. This study gradually introduced the relevant AR module design, incorporated CT teaching methods into the AIoT course, and analyzed the effect of the AR modules on students' learning intentions and CT competences. Finally, we present a discussion and elaboration on the research based on the relevant data measurements.

## Related Literature

### Computational Thinking

CT first appeared in 1980 when Seymour Papert proposed a deeper contemplation on computers and suggested incorporating the changes computers have caused into children's learning and self-expression. The concept of CT was mentioned again in 1993 (Papert, 1980, 1993); however, it did not receive much attention until 2006 when Wing clarified the concept and propagated its application to problem solving. In his later research, Wing called on all educational research fields to emphasize CT as a core skill for K-12 education and develop a CT pedagogy (Wing, 2006, 2008, 2011). CT competence can be divided into the following four categories: decomposition, pattern recognition, pattern generalization and abstraction, and algorithm design. Wing (2008) pointed out that CT competency can be acquired through the successive cultivation of skills in the four aspects. Since then, CT has attracted growing attention from educators and education researchers and is considered a critical ability that enables students to grasp basic problem-solving skills (Qualls and Sherrell, 2010; Weintrop et al., 2016). In K-12 education, students' CT competency is mainly fostered through programming training (Goyal et al., 2016; Wei et al., 2020). CT can help students attain higher-level thinking processes, such as problem decomposition and innovative thinking (Barr and Stephenson, 2011; Shute et al., 2017). In addition to K-12 education, CT is also applicable to the field of higher professional education (Tang et al., 2020). In professional fields, CT can help with problem solving and analysis, further enhancing programming learning motivation. For students in nonprofessional information fields, CT can facilitate their understanding of how programming works and further enhance their interest in programming skills (Aoki et al., 2013). Through different courses, CT teaching can foster skills in various fields, such as mathematics, robotics, and music, and even integrate current IoT applications with AI technology learning (Benakli et al., 2017; Bell and Bell, 2018). Many studies have also developed relevant technological tools to support CT learning, such as augmented reality (AR) technology, virtual reality (VR) technology, and even robotic aids (Weintrop et al., 2014; Atmatzidou and Demetriadis, 2016; García-Valcárcel-Muñoz-Repiso and Caballero-González, 2019; Lin and Chen, 2020).

### AR Technology

The key concept of AR is to expand the reality field. With the introduction of virtual information and objects, AR can strengthen the understanding of relevant information technology or enhance the understanding of a given object. AR was first proposed as part of the reality–virtuality continuum by Milgram and Kishino (1994), who defined its theoretical foundation. AR consists of virtual and real environments, real-time interactive interfaces, and an overall environment of spatiality. The application of AR technology can strengthen users' perception of real objects and their interaction with virtual data. AR technology is widely used in engineering, science, humanities, medicine, and other fields due to its ability to augment information and overlay it on real scenes to strengthen users' comprehension and cognition (Van Krevelen and Poelman, 2010). In the educational field, advances in information technology have facilitated a transition from traditional face-to-face teaching to computer-aided teaching, and AR is widely used to assist students in conceptual learning (Bacca et al., 2014; Akçayir and Akçayir, 2017). Radu (2014) conducted a comprehensive review of 26 publications and produced a list of positive and negative effects of educational AR technology on students. Radu concluded that AR is beneficial to enhancing students' motivation, promoting collaboration among students, developing spatial abilities, and improving physical task performance. As for its negative effects, Radu noted that AR places an additional cognitive burden on students and can cause usability issues. However, the novelty of and feedback from interactions with AR information can increase students' interest in learning. Furthermore, AR allows students to directly interact with objects and scenes, which is difficult for traditional teaching tools to achieve. For abstract scientific concepts, in particular, AR can effectively improve understanding and build self-confidence in learning. Thus, using AR as a teaching tool can help students with scientific exploration and provide relatively unique educational benefits (Cheng and Tsai, 2013; Soltani and Morice, 2020). In AR, the interaction between virtuality and real scenes can facilitate students' interactive exploration of information. Hence, it is especially useful in the fields of science, technology, engineering, and mathematics (STEM) with respect to spatial abilities, practical skills, conceptual understanding, and scientific inquiry and learning (Ibáñez and Delgado-Kloos, 2018; Phupattanasilp and Tong, 2019).

## Research Framework and Design Methods

### Research Model

**Figure 1:**
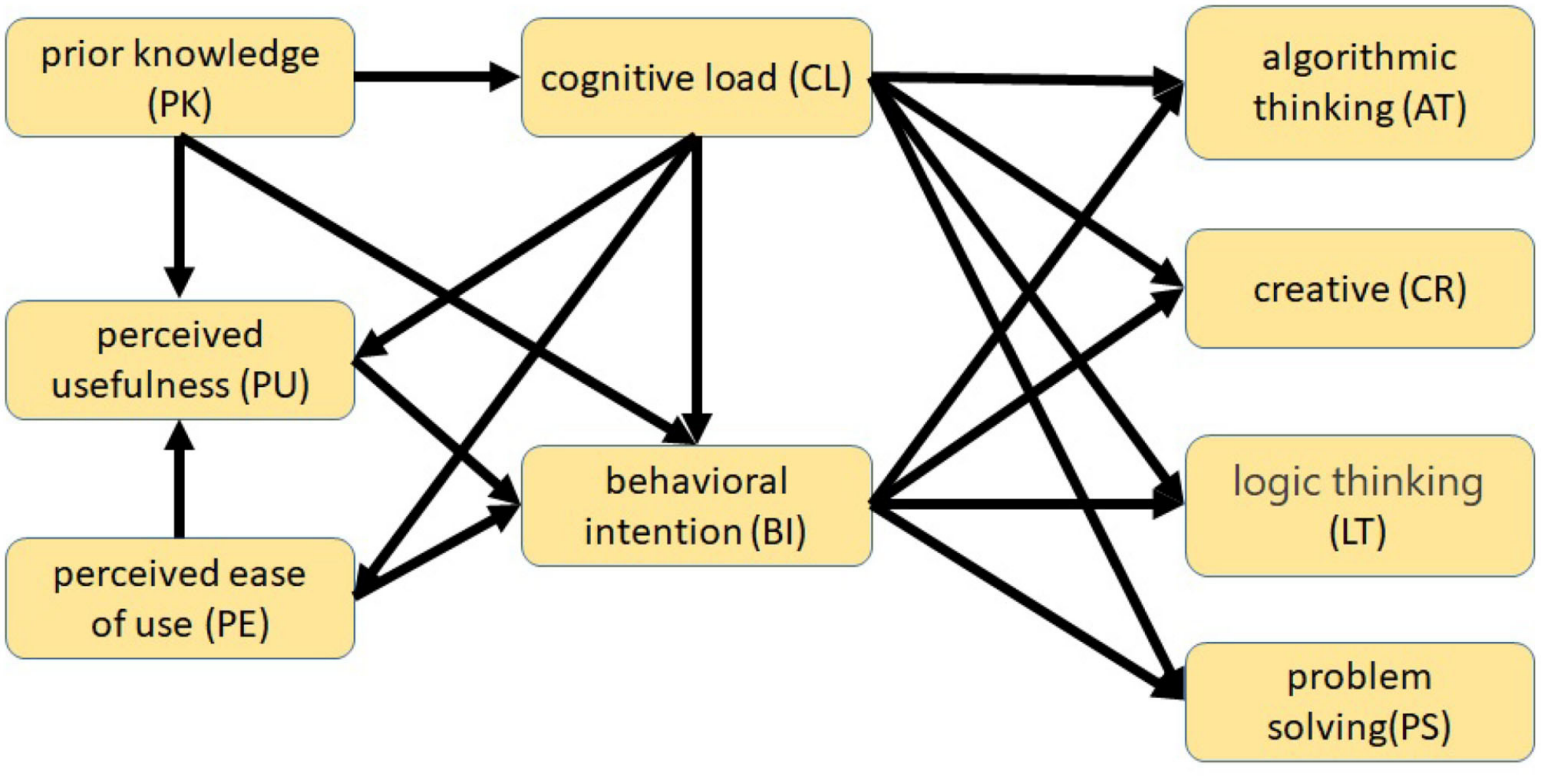
Effect of introducing AR IoT on the outcome of CT competency training.

This study aimed to examine students' CT competency performance in AIoT courses with the aid of AR IoT applications. The overall research model is shown in Figure 1. This study referred to and modified the technology acceptance model to investigate students' intention to use AR IoT applications (Lijnse, 1995; Méheut and Psillos, 2004). The technology acceptance model is based on the theory of reasoned action and has inherited the essence of reasoned action. It posits that belief perceptions affect attitudes, which, in turn, affect behavioral intention; behavioral intention can have a significant and positive effect on system use. The technology acceptance model proposes two factors that can affect acceptance among information system users, namely, perceived usefulness and perceived ease of use. These two cognitive factors are considered to correspond to users' evaluation of performance and effort. The technology acceptance model can facilitate our exploration of the factors that influence system use. Perceived usefulness and perceived ease of use are both subject to the influence of external variables, i.e., factors related to the system or teaching model. In addition, this study introduced students' prior knowledge of IoT and AR, considered the effects of AR cognitive load on usage intention (Radu, 2014; Chang and Chen, 2018), and explored whether these factors can influence CT competency performance.

### AIoT AR Application Design

**Figure 2:**
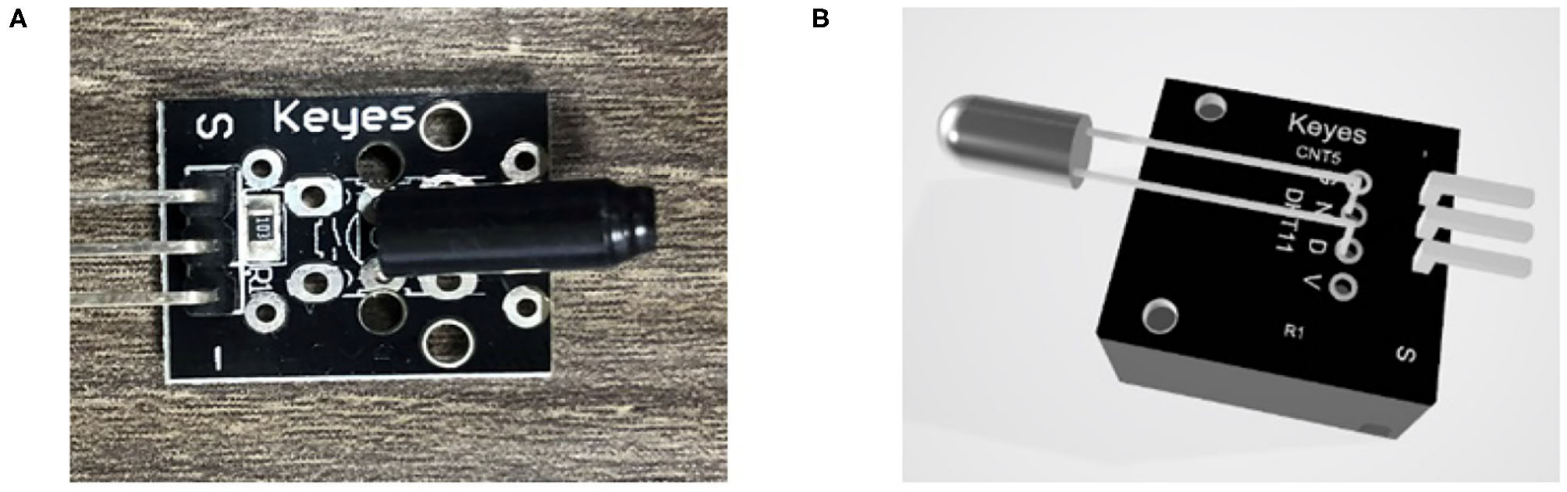
**(A)** Sensor module and **(B)** 3D AR sensor module.

The main objective of this study is to design an AIoT AR application and introduce it to the AIoT teaching environment to provide students with diverse knowledge and modes of thinking when they encounter overly complex AIoT scenarios with a wide range of considerations, so as to cultivate CT competency. First, this study used the Unity program and referred to the IoT sensor module kit to create a total of 37 IoT sensor modules, as shown in Figure 2.

**Figure 3:**
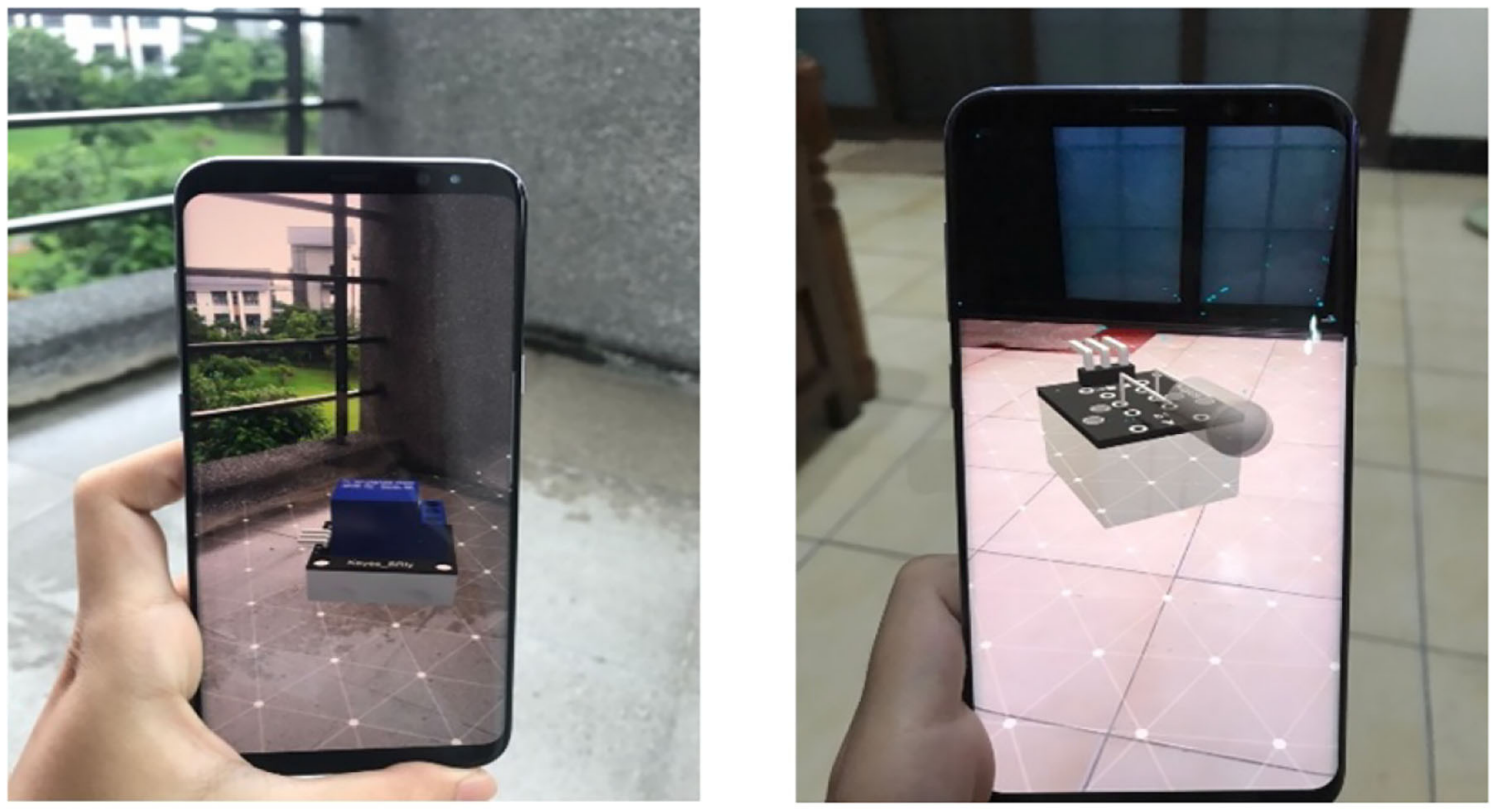
AR module with ARcore.

In order to ensure that the AIoT AR module can be overlaid on application scenarios, ARCore was mainly used for preliminary construction. ARCore is an AR development platform launched by Google; it includes a set of new application programming interface (API) and frameworks. It combines a camera, an inertial measurement unit (IMU), a three-dimensional accelerometer, and a gyroscope (known as “sensor fusion”) to obtain feature points and point cloud data in order to track certain points in different visual fields and attempt to identify their locations in reality. After acquiring their locations, simultaneous localization and mapping (SLAM) is incorporated to help ARCore locate the user (device) and identify the objects around the user. Once the localization is completed, each frame is compared with the previous frame in the photographic image to identify similar points in order to confirm the user's relative distance and displacement distance, so as to ensure that the device's localized position maintains a relative distance in movement. The AR module as a whole can be imported into the application, as shown in Figure 3. The use of AR in the AIoT course provides a novel way with learning and thinking. The student not only views the 3D image of the sensor but tries to plan and deploy through 3D AR sensors in actual field scenes. Compared with the traditional way of thinking through pen and paper or slides, the AR can help students more easily think about problem solving and improve AIoT practice implementations.

### Introducing the AR Application Into CT Planning

**Figure 4:**
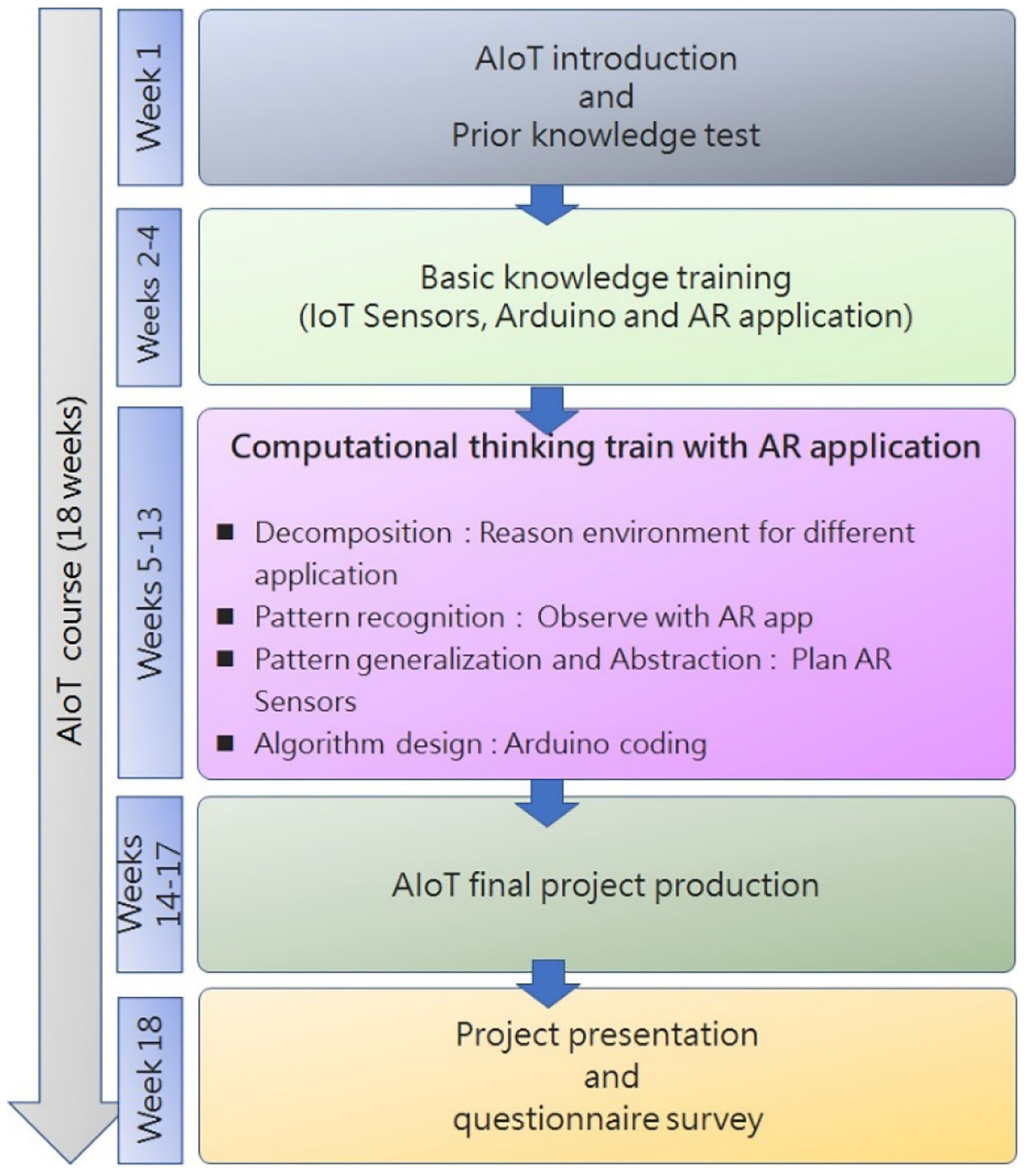
AIoT curriculum.

In this study, students who took the AIoT application practical courses were recruited as participants to help establish an AIoT curriculum. The curriculum plan is shown in Figure 4.

Week 1 of the 18-week course introduced AIoT and the learning objectives. Students took a prior knowledge test on AIoT and AR to evaluate their preexisting abilities. Weeks 2–4 introduced sensor components, IoT Arduino coding, and AR application operation methods. In Weeks 5–13, students were divided into groups for three sessions of CT training. Each group used fixed sensors for various applications. Based on considerations of the scenarios' accessibility to the students, the selected applications included smart agriculture, smart homes, smart campuses, smart lighting, and smart transportation. Each training cycle lasted 3 weeks. In the first of each training cycle, students were asked to study the problems in the actual application environment, think about the problem in the context of AR applications, and select suitable AR sensor modules for placement design. In the second week, students were asked to think about the algorithms and coding required for each sensor based on the AR design plan. In the third week, each group was asked to present their report. As part of our research objectives, the AR module was used to teach AIoT courses in order to foster CT competence in the following four categories.

In the final specifying weeks of the course, each student used sensors to solve related problems independently. At the end of the semester, internal and external reviewers were invited for the project review. Each final project presentation was reviewed by an advisor and two professional industry experts. The rubric for the review was based on CT competences, including algorithmic thinking, creativity, logical thinking, and problem-solving skills. The total score was used as an indicator of the students' personal learning feedback and motivation. Higher scores indicated stronger learning motivation, and vice versa.

## Experimental Analysis

### Data Collection

A total of 96 IoT students from two classes were invited to participate in the experiment. A total of 91 students completed the course and filled out the survey at the end of the semester. Due to the small number of participants, partial least squares structural equation modeling (PLS-SEM) was used to analyze the data (Henseler et al., 2009; Hair et al., 2014). This study's sample size was based on the 10-times rule, which proposes that 90 samples are required to investigate nine indicators (Chin and Newsted, 1999).

### Data Collection

SmartPLS was used to analyze the relevant data from the survey. Data for survey items PU3 and BI2 were excluded because their factor loadings were below 0.7. The PU3 item is that I can shorten the time of learning AIoT with AR apps, and the BI2 item is that I am willing to spend some time in AR application to learn AIoT technology. Through detailed interviews, it is found that the main reason is that some students believe that the BI2 item requires extra time to study after class, which results in answer errors. Moreover, some students do not agree with AR corresponding to shortening the learning time according PU3. The results of the subsequent analysis are shown in Table 1. All items had a Cronbach's alpha and rho_A >0.7; the composite reliability (CR) of prior knowledge and cognitive load was slightly <0.7 but still within an acceptable range (Fornell and Larcker, 1981). The average variance extracted (AVE) for all items was >0.5, satisfying the criteria for the convergence validity of variable variances.

**Table 1:** Construct validity analysis.

	**Cronbach's alpha**	**rho_A**	**CR**	**AVE**
PK	0.713	0.724	0.682	0.624
PU	0.814	0.805	0.812	0.614
PE	0.751	0.812	0.802	0.754
CL	0.702	0.713	0.681	0.620
BI	0.852	0.852	0.715	0.574
AT	0.913	0.892	0.845	0.752
CR	0.721	0.745	0.785	0.542
LT	0.851	0.881	0.892	0.621
PS	0.749	0.785	0.712	0.674

The discriminant validity of each construct was evaluated using the heterotrait–monotrait ratio (HTMT), and the results are shown in Table 2. All constructs met the requirements for correlation discriminant validity, at <0.9 (Henseler et al., 2016).

**Table 2:** HTMT of each construct.

	**PK**	**PU**	**PE**	**CL**	**BI**	**AT**	**CR**	**LT**	**PS**
PK									
PU	0.851								
PE	0.525	0.612							
CL	0.641	0.415	0.528						
BI	0.645	0.542	0.428	0.745					
AT	0.741	0.852	0.314	0.641	0.514				
CR	0.645	0.841	0.745	0.854	0.486	0.514			
LT	0.558	0.765	0.354	0.456	0.487	0.745	0.654		
PS	0.584	0.648	0.854	0.674	0.645	0.645	0.548	0.674	

**Figure 5:**
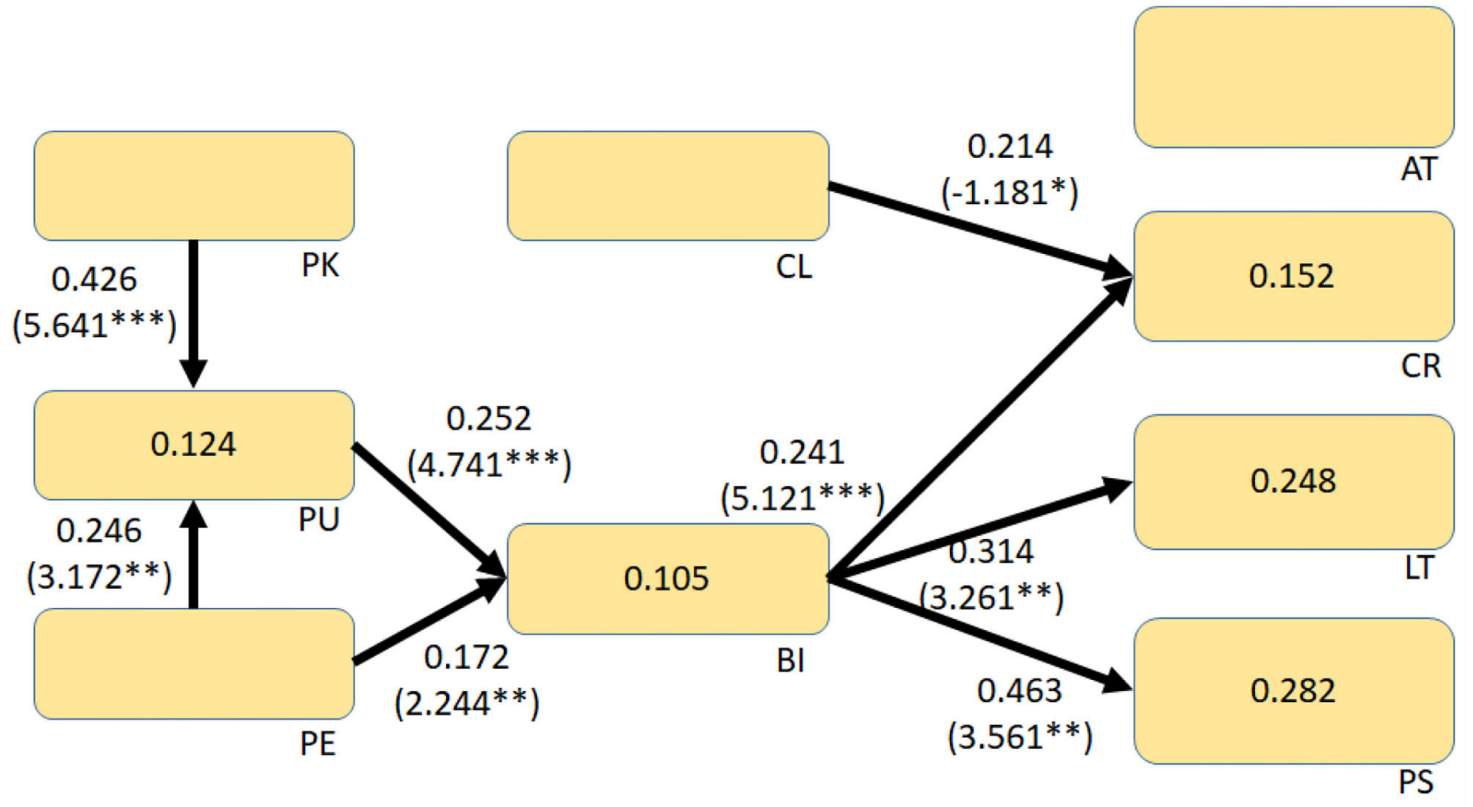
Structural equation model. *p < 0.05, **p < 0.01, ***p < 0.001.

Bootstrapping, with 5,000 iterations, was then used to calculate the *t*-value, *p*-value, and *R*^2^-value. The overall structure diagram is shown in Figure 5. According to the structural equation, students' intent to use the AR AIoT application was influenced by perceived usefulness and perceived ease of use. The overall *R*^2^ value is 0.105, but the overall effect was small and was not affected by the technological cognitive load. Furthermore, students' intentions had corresponding effects on their CT competency performance in creativity, logical thinking, and problem solving. Thus, the results show that the introduction of AR AIoT into the course had a significant impact.

## Discussion and Study Limitations

This study attempted to an AIoT course, aiming to integrate an AR application and CT competency to help students foster problem-solving skills and omitting creativity. This research model mainly explores the impact of the proposed AIoT learning on students' cognition, use intention, and CT skills under different prior knowledge and cognitive load situations. The following conclusions were drawn based on our results:

1. The proposed AIoT learning impacted CT competency performance.
  According to the results of the analysis, the introduction of the AR AIoT application designed in this study had an impact on CT competency performance in the areas of creativity, logical thinking, and problem solving. This shows that compared to having students think through problems in a regular classroom setting, the AR AIoT application allowed students to understand problems within actual scenarios and further refine overall application planning through the placement of AR sensors. The prior knowledge mainly explores the influence of students on the overall AR use intention to varying degrees. Perceived ease of use mainly explores whether students are easy to use AR applications, and its main impact is the design of AR applications without prior knowledge. However, prior knowledge is only relevant to cognitive usefulness but not to cognitive load and learning motivation. According to the results of the detailed interviews, some students believe that their main influence is on the ease of use of the application, and this application will continue to modify the user interface to improve the intention of use.
2. The AR application had No impact on cultivating students' algorithmic thinking of CT skills.
  Among the CT competences, algorithmic thinking mainly corresponds to programming and algorithm selection skills. In contrast, although the AR application can help students understand problems, at the current stage, it is still necessary to use computer programming for course coding exercises. Therefore, in this regard, the application does not improve the effectiveness of students' programming skills or algorithm exercises.
3. The technological cognitive load had little effect on students.
  In this study, it was originally hypothesized that the cognitive load of science and technology may affect students' usage intentions and learning outcomes (Radu, 2014). However, according to the experimental results, no effect was found on usage intention. The influence on CT competences was limited to negative effects on creativity. Thus, it can be speculated that at the current stage, this study mainly tested participants from the information discipline who had a certain level of prior knowledge and familiarity with AR applications; hence, the effect of this factor is not significant.
  Since the course used in this study is a course for information technology professionals, the study's results cannot be extended to the AR application's impact on usage intention and cognitive load in a non-information professional context. Future studies should attempt to help students cultivate algorithmic thinking using relevant technological approaches and conduct tests in non-information education fields.

## Data Availability Statement

The original contributions presented in the study are included in the article/supplementary material, further inquiries can be directed to the corresponding author/s.

## Author Contributions

Y-SL proposed the whole research model theory and literature research. S-YC design 3D AR models and mobile apps. C-WT collect questionnaire data and analysis. Y-HL carried out course teaching and experimental design. All authors contributed to the article and approved the submitted version.

## Conflict of Interest

The authors declare that the research was conducted in the absence of any commercial or financial relationships that could be construed as a potential conflict of interest.
